# Supplementary material for: Adrenal suppression in patients with chronic obstructive pulmonary disease treated with glucocorticoids: Role of specific glucocorticoid receptor polymorphisms
Source: PLoS One. 2022 Feb 4;17(2):e0262898. doi: 10.1371/journal.pone.0262898 (PMC8815987; doi:10.1371/journal.pone.0262898)
Supplement: Attachment — Submitted filename: point_point_response_11SEP2021.docx [file pone.0262898.s001.docx]

Dr. Emily Chenette

Editor-in-Chief

*PLOS ONE*

**Manuscript reference number: PONE-D-21-06570**

11 September 2021

Dear Dr. Chenette,

Thank you for the invitation to submit a revised version of our paper:

*PONE-D-21-06570 ‘Adrenal suppression in patients with chronic obstructive pulmonary disease treated with glucocorticoids: role of specific glucocorticoid receptor polymorphisms.*

We would like to thank the reviewers and editors for their valuable comments. We have revised the paper according to these comments, and we believe it has been substantially improved. Below are our point-by-point responses to the comments made by the reviewers. The reviewers’ comments are listed ‘C1, C2, C3, etc.’ and our responses are marked ‘R_C1, R_C2, etc.’

On behalf of the authors,

Pradeesh Sivapalan MD PhD and

Professor Jens-Ulrik Jensen MD PhD

EDITOR

__________________________________________________________________

**C1:** *Please ensure that your manuscript meets PLOS ONE’s style requirements, including those for file naming.*

**R_C1:** We have now ensured that our manuscript meets the PLOS ONE style requirements as requested.

Changes include:

- Double spacing
- Genes and mutations are now in italics
- Figure captions have been inserted in the manuscript
- Correct citing of figures (e.g. ‘Fig 1’ instead of ‘Figure 1’)
- Reference style is rectified
- Spell-checking

__________________________________________________________________

**C2:** *We note that you have indicated that data from this study are available upon request. PLOS only allows data to be available upon request if there are legal or ethical restrictions on sharing data publicly.*

**R_C2:** Unfortunately, we are not allowed to share de-identified data sets without permission. We have explained the reasons for this in our cover letter for this resubmission. However, we will do everything possible to assist researchers who wish to obtain these data from the Danish authorities.

__________________________________________________________________

Reviewer #1: Manuscript number: PONE-D- 21-06570

Adrenal suppression in patients with chronic obstructive pulmonary disease treated with glucocorticoids: role of specific glucocorticoid receptor polymorphisms

Sivapalan and co-workers investigated the possible association between single-nucleotide polymorphisms (SNPs) of glucocorticoid receptor (GR) and metabolic disorders in patients with chronic obstructive pulmonary disease (COPD). In particular, the authors focused on the connection between GR haplotypes and adrenal suppression in patients with COPD following administration of systemic glucocorticoid (therapy for COPD exacerbation treatment). The authors conclude that COPD patients treated with systemic GC for their exacerbation and characterized by BclI and N363S gene polymorphisms did not have a major risk of acute adrenal suppression or metabolic disorders.

It is an interesting study, but the following should be addressed:

*Major comments:*

__________________________________________________________________

**C1:** *The author in the Material and Methods section not reported the primer sequence used for genotyping the four GR polymorphisms. Please provide this information; it is useful for the readers that want to perform similar analysis.*

**R_C1:** Thank you for this suggestion. We have revised the Materials and Methods section and included the primer sequences in section 2.2:

*Sequences of primers used in PCR*

| *Polymorphism* | Forward | Reverse |
| --- | --- | --- |
| *Bcl*I | TGCACCATGTTGACACCAATTCCT | GGTCTTGCTCACAGGGTTCTTGCC |
| *N363S* | TCATCGAACTCTGCACCCCTGG | AGTTGTCATCTCCAGATCCTTGGCA |
| *9β* | CCTACGCAGTGAAATGTCAGACTGT | TGCCAATTCGGTACAAATGTGTGGT |
| *ER22/23EK* | AACCCCAGCAGTGTGCTTGC | AGCGACAGCCAGTGAGGGTGA |

__________________________________________________________________

**C2:** *Please provide a new table with the clinical, functional and biologic characteristics of all 77 patients included in the study: i.e. the prevalence of male and female, COPD step based on GOLD guidelines, age, BMI, etc.*

**R_C2:** Thank you for this suggestion. We have now added clinical, functional and biologic characteristics of all patients to Table 1, including sex, GOLD class, age, and BMI.

__________________________________________________________________

**C3:** *on Results section authors affirm that: “the ischaemic heart disease, which seemed more prevalent in the low plus wild type (25%) than 139 in the high (3%) GC-sensitivity group.” In my opinion, it is necessary to report this statistical significance in Table 1. This could be misleading for the readers.*

**R_C3:** We agree and have now included *p*-values in Table 1 for all comorbidities, medication, and GOLD class.

__________________________________________________________________

**C4:** *Page 4 lines 131-134 authors underline that: “We found a negative association between male gender and stimulated cortisol response ( Rho = -0.24, p = 0.042), but no correlation to other possible confounders: BMI (Rho = -0.15, p = 0.22), age (Rho = 0.06, p = 0.63), accumulated GC use 6 months before test (Rho = -0.14, p = 0.23) and days since completed GCs (Rho = 0.09, p = 0.47).” Please provide a table or figure that show these findings*

**R_C4:** Thank you for this suggestion. We have now provided a table showing these findings. Please see below also:

**Table 2.** Relationship between stimulated cortisol response and possible confounders.

|  | **Spearman’s Rho** | ***P*-value** |
| --- | --- | --- |
| Male | –0.24 | 0.042 |
| BMI | –0.15 | 0.22 |
| Age | 0.06 | 0.63 |
| Accumulated GC use 6 months before test | –0.14 | 0.23 |
| Days since completed GCs | 0.09 | 0.47 |

*Minor comments:*

__________________________________________________________________

**C1:** *Page 2, line 65: Provide reference.*

**R_C1:** Thank you for noticing this mistake. Page 2, line 65 has now been updated with a reference.

__________________________________________________________________

**C2:** *The authors did not indicate the inhaled corticosteroid considered for the analyses (beclomethasone or fluticasone). Please provide this information.*

**R_C2:** Thank you for this comment. This has now been clarified in the methods section as follows:

“2.2. Exposure to ICS

All ICS prescriptions (alone or in a combination inhaler) prior to cohort entry were identified. These included: beclomethasone, budesonide, fluticasone, ciclesonide, and mometasone. All doses of ICS were converted to budesonide-equivalent doses: Beclomethasone and mometasone were considered equivalent to budesonide. Fluticasone propionate and ciclesonide were converted to budesonide doses using ratios of 2:1 and 2.5:1, respectively.”

__________________________________________________________________

**C3:** *Page 3, line 97: replace the square with the symbol β.*

**R_C3:** Thank you for pointing out this mistake. The square has now been replaced with β.

__________________________________________________________________

**C4:** *Page 8, lines 225-227: consider moving the sentence in the “Corticotropin test” (Materials and Methods section).*

**R_C4:** Thank you for this suggestion. This sentence has now been moved to the method section, page 3, line 83:

*“Also, ICS treatment was paused 24 hours before the corticotropin test to avoid cross-reactivity with cortisol in the assay.”*

__________________________________________________________________

Reviewer #2: In the present study, Sivapalan et al evaluate the role of glucocorticoid receptor polymorphisms in the development of adrenal suppression following ICS treatment for COPD exacerbations.

**C1:** As rightly pointed out in the discussion, uniting subjects with low-sensitive haplotypes with mixed ones (wild-type) does not make particular sense, if not to increase the numerosity. In this way, however, the real comparison between low-sensitive and high-sensitive is lost. In fact, I would expect wild-types to have an intermediate behavior between low- and high-sensitive haplotypes.

**R_C1:** As the reviewer notes, combining low-sensitivity GR gene haplotypes with wild-type haplotypes is a limitation of our study. However, separate analyses of each of the SNPs from all 78 patients also found no correlation. Therefore, it is unlikely that a large effect has been overlooked. We have elaborated on this issue in the discussion:

“Unfortunately, we were unable to obtain the required number of patients with prespecified haplotypes that was suggested by the power calculation. Combining low-sensitivity GR gene haplotypes with wild-type haplotypes is a limitation of our study. Had we only included low-sensitivity GR gene haplotypes, we would have required many more patients. We decided that it was unnecessary to include more patients to test our hypothesis. We acknowledge that having low-sensitivity and high-sensitivity GR gene haplotype groups and excluding patients with wild-type haplotypes would have been optimal. However, separate analyses of each of the SNPs from all 78 patients also found no correlation. Therefore, it is unlikely that a large effect has been overlooked. Nonetheless, our results will need to be validated by larger studies in the future. In addition, our cohort included many older patients with substantial comorbidities that may mask the effects of GR polymorphisms. Further studies, involving *ex vivo* models and reproduced alleles on plasmids, may validate the study rationale. ”

*Comments*

**C2:** How many subjects are actually with low-sensitive haplotypes and how many are wild-type? Furthermore:

**R_C2:** Thanks for this comment. There were 76% with the high-sensitivity genotype, 4% with the low-sensitivity genotype, and 20% with the wild-type genotype. This is now described in the results section.

*“In the haplotype analyses, we included the 48 patients with the high-sensitivity (76%) or low-sensitivity (4%) GR gene haplotypes or the wild-type GR haplotype (20%) (Table 3)”*

__________________________________________________________________

**C3:** *The authors enrol 77 patients, but the description in table 1 is only about 50. What characteristics do these additional 27 have and why were they excluded?*

**R_C3:** The additional patients you describe had a mixture of high- and low-sensitivity GR haplotypes. Therefore, these patients were excluded from the analyses that focused on haplotypes. This is now described in the Methods section.

*“We divided patients into an expected high-sensitivity GR gene haplotype group (patients carrying Bcl1 and/or N363S, but not ER22/23EK and 9β) and an expected low-sensitivity (patients carrying ER22/23EK and/or 9β, but not Bcl1 or N363S) plus wild-type (patients who exhibited wild-type genotypes for all four polymorphisms) GR gene haplotype group. Patients who had a mixture of high-sensitivity and low-sensitivity genotypes were excluded from the haplotype analyses.”*

__________________________________________________________________

**C4:** *What is the real distribution of haplotype sets in the population? The individual SNPs are instead reported.*

**R_C4:** Thank you for this comment. We have included this in abstract and results**: “**In the haplotype analyzes, we included the 48 patients with either high- (76%) or low-sensitivity GR gene haplotypes (4%) or the wild-type GR haplotype (20%).”

__________________________________________________________________

**C5:** *In estimating the sample size, how do the authors define the reported corticotropin values and why do they choose a 3:1 ratio?*

**R_C5:** Thanks for this comment. Our sample size calculation was based on studies from patients with rheumatoid arthritis. This is now described in detail in the statistical analysis section.

*“The sample size calculation was designed for the haplotype analyses. We performed a* t*-test in which the ratio among the groups was 3:1* *because previous results from other cohorts suggested that patients would exhibit this distribution of haplotypes [19]. The mean difference in cortisol concentration between patients with the wild-type plus ER22/23EK and/or 9β polymorphisms and those with the homo/heterozygous Bcl1 and or N363S polymorphisms was set to 100 nmol/L. These parameters were based on data from two previous rheumatology studies [11, 20].”*

__________________________________________________________________

**C6:** *In the materials and methods it is not clear how the continuous data are expressed.*

**R_C6:** Thank you for this comment. The relevant part of the statistical analysis section now reads:

“Continuous data were expressed as means with standard deviations when normally distributed or as medians with interquartile ranges when non-normally distributed. Categorical variables were expressed as counts and percentages.”

__________________________________________________________________

**C7:** *In Table 3 and Figure 2, the authors found an inverse association between ICS and P-cortisol only in high-sensitive subjects (which are 2.5 more numerous than low + wild types).*

**R_C7:**

After we converted all ICS to budesonide equivalents, there was little change in the results: “Carriers of the high-sensitivity GR gene haplotype exhibited a strong inverse relationship between inhaled corticosteroid (ICS) dose and stimulated P-cortisol concentrations (slope, –1.35 vs. 0.96; *p* = 0.17; Table 5, Fig 2), compared to patients with the low-sensitivity haplotype plus those with wild-type haplotypes.”

__________________________________________________________________

**C8:** *Figure 1 shows a value probably to be considered an outlier.*

**R_C8:** Thanks for making us aware of this. We have deleted the outlier and created a new figure.

__________________________________________________________________

**C9:** *Tables are not numbered consecutively*

**R_C9:** Thank you for noticing this mistake. We have numbered the tables consecutively.

__________________________________________________________________

**C10:** *There are typos*

**R_C11:** Thank you for pointing this out. The manuscript has now been thoroughly proofread to eliminate typos. Spelling has been changed from British English to American English.

**C12:** *The work is interesting, however, it should be re-evaluated with a better subdivision of the groups.*

**R_C12:** Thanks for all the suggestions. We have revised the manuscript based on the reviewers’ comments and believe it has been substantially improved.
