# Supplementary material for: Adrenal suppression in patients with chronic obstructive pulmonary disease treated with glucocorticoids: Role of specific glucocorticoid receptor polymorphisms
Source: PLoS One. 2022 Feb 4;17(2):e0262898. doi: 10.1371/journal.pone.0262898 (PMC8815987; doi:10.1371/journal.pone.0262898)
Supplement: Attachment — Submitted filename: response_to_reviewers_10DEC2021.docx [file pone.0262898.s002.docx]

Dr. Emily Chenette

Editor-in-Chief

*PLOS ONE*

**Manuscript reference number: PONE-D-21-06570**

10 December 2021

Dear Dr. Chenette,

Thank you for the invitation to submit another revised version of our paper:

*PONE-D-21-06570 ‘Adrenal suppression in patients with chronic obstructive pulmonary disease treated with glucocorticoids: role of specific glucocorticoid receptor polymorphisms.*

We would like to thank the reviewers and editors for their valuable comments. We have revised the paper once more according to the comments by Reviewer #2, and we believe it has been substantially improved. Below are our point-by-point responses to the comments made by the reviewers. The reviewers’ comments are listed ‘C, CA, CB, etc.’ and our responses are marked ‘R_C, R_CA, R_CB etc.’

On behalf of the authors,

Pradeesh Sivapalan MD PhD, Stina Borresen MD PhD, Marianne Klose MD PhD and Professor Ulla Feldt Rasmussen & Professor Jens-Ulrik Jensen MD PhD

Reviewer #1

**C:** The authors accurately reviewed the manuscript and is now considered to be suitable for publication in Plos One.

**R_C:** Thank you, we are pleased to hear so.

Reviewer #2

*This reviewer’s comment has been split up in sections and answered accordingly.*

**C-A:** Sivapalan and collaborators have significantly improved their manuscript over the previous version. However, this version still had pitfalls and unclear passages, albeit of minor importance.

**R_C-A:** Thank you for this comment. We have made substantial changes to clarify several passages, as seen in the new revised manuscript.

**C-B:** […] Why did the number of enrolled patients go from 77 to 78? In the description of the numerosity calculation, the authors wrote: "16 and 48 patients were included in each of the two groups (i.e., 64 patients in total). Assume that 20% of the recruited patients would not be placed in either of the two groups, additional 13 patients were needed, resulting in recruitment of 78 patients to the study". However, adding 13 to 64, the result is 77, as in the previous version of the manuscript, and not 78.

**R_C-B:** Thank you for this important comment. Unfortunately, we made a mistake in declaring 77 enrolled patients in the original manuscript. The correct number is 78. Also, it was unclear that the assumption was approximately 20% (i.e. 18%), thus resulting in 14 patients expected not to be placed in either group. It has now been clarified that the 14 additional patients enrolled was a decision based on the approximate assumption, and not exactly equal to the assumed. It now reads as follows:

Page 6, lines 144-146:
*“Assuming that approximately 15-20% of the recruited patients would not be placed in either of the two groups, additional 11-16 patients were needed, resulting in a decided recruitment of 78 patients to the study.”*

**C-C:** […] Furthermore, a posteriori, it would have been helpful to predict the non-usability of data from "mixed genotype" subjects to recruit more individuals.

**R_C-C:**

Yes, we agree with the reviewer. Unfortunately, there was sparse data on COPD patients, so it was difficult to predict this in our power calculation. We must be aware of this in future studies in this area.

**C-D:** […] Had the authors designed the used primers? If so, how were primers designed? Otherwise, what is their source?

**R_C-D:** Yes, the primers were designed by the authors. This, and the design of the primers, has now been clarified in the revised manuscript. It now reads as follows:

Page 5, lines 121-122:

“Primers were designed using Primer-BLAST [16] and ordered through Eurofins[17].”

**C-E:** […] In the text, the descriptions of tables 3 and 5 are missing.

**R_C-E:** Thank you for this comment. We have now described table 3 and 5 in the manuscript. Please see below:

Page 9, lines 182-184:

*“There was no correlation between accumulated systemic glucocorticoids and 30 min cortisol concentrations in the high-sensitivity haplotype group (p-value, 0.79) or the low-sensitivity plus wild-type haplotype group (p-value, 0.26) (Table 3).”*

Page 10, lines 201-204:
*“Carriers of the high-sensitivity GR gene haplotype exhibited a steeper decline in stimulated P-cortisol with increased ICS dose compared to patients with the low-sensitivity haplotype plus those with wild-type haplotypes (slope, –1.35 vs. 0.94; p = 0.17; Fig 2 and Table 5).”*

**C-F:** […] Furthermore, given the small population, it would have been interesting to evaluate the characteristics of the "mixed genotype" population. Would intermediate features be conceivable, like wild-type, or would one component prevail?

**R_C-F:** Thank you for this valuable comment. Characteristics of the mixed genotype population has now been added to Table 1. No significant differences were observed. Mixed type has also been included in Table 3 and Table 5, as well as the total cohort. We agree it would be of great interest to analyse specific haplotypes in the mixed type group as well as the low- and wild groups individually. Power for such analyses, would though require a larger cohort, as multiple haplotypes are present in those groups.

This has now been commented on in the discussion in the manuscript (page 14, lines 300-302):

*“Also, it would have been interesting to consider the effect of the different haplotypes present in the mixed genotype and wild type group, but this was not possible with the obtained study sample size.”*
